# Cross-sectional nutrition assessment in a refugee camp in Gambella region, Ethiopia

**DOI:** 10.1093/eurpub/ckac131.518

**Published:** 2022-10-25

**Authors:** R Benoni, A Sartorello, E Paiola, F Moretti, R Buson, A Tsegaye, S Tardivo, F Manenti

**Affiliations:** 1 Department of Diagnostics and Public Health, University of Verona, Verona, Italy; 2 Doctors with Africa CUAMM, Padua, Italy

## Abstract

Minors account for 20 percent of the world’s migrants, reaching 33 million in 2019. The prevalence of malnutrition has been reported between 17 and 21% among refugees. However, data about Sub-Saharan African refugees is lacking. The study evaluates the nutritional status of refugees in the Nguenyyiel camp in Gambella (Ethiopia). The retrospective cohort study included all children under five attending the first visit to the refugee camp’s health post between 01/06/2021 and 31/08/2021. Sociodemographic data, body weight, and upper arm circumference (MUAC) were recorded. The z-score of weight for age (WFA) and MUAC for age (MUACZ) were estimated using the R ‘anthro’ package developed by the World Health Organization (WHO). Children with WFA <-2 standard deviations (SD) were considered underweight, those >2SD overweight. A MUACZ <-2SD defined acute malnutrition. Among the 782 patients admitted, 415 (53%) were under five. Females were 195 (47%). The mean age was 2.1 years (SD 1.6). The mean body weight was 11kg (SD 5). Considering the WFA, 200 (48%) children were within +2 SD. Children with WFA <-2SD were 92 (29%), those > 2SD were 28 (9%). The frequency of children with WFA <-2SD was higher in boys (p = 0.049). There were no differences in the frequency of children with WFA >2SD based on sex (p = 0.998). WFA decreased as age increased (p = 0.048). MUAC was recorded for 273 (66%) children. The mean MUAC was 14.2mm (SD 2.4). Children with MUAC z-scores within +2SD were 245 (77%). Children <-2SD were 92 (8%). The frequency of children with MUACZ <-2SD was not significantly different based on sex or age (p = 0.125, p = 0.324). The prevalence of malnutrition was moderate in the Nguenyyiel camp. At the same time, the frequency of underweight children was high, particularly among boys (34%) and with increasing age.

**Key messages:**

• Nutrition remains a problem in refugee camp settings, especially in children.

• Ensuring the health of refugees, as vulnerable population, should be a priority for both governments and international organizations.

